# Multimodal Analgesia in Paving the Way for Enhanced Recovery After Cardiac Surgery

**DOI:** 10.21470/1678-9741-2022-0058

**Published:** 2023

**Authors:** Rohan Magoon, Jes Jose

**Affiliations:** 1 Department of Cardiac Anaesthesia, Atal Bihari Vajpayee Institute of Medical Sciences (ABVIMS) and Dr. Ram Manohar Lohia Hospital, New Delhi, India.; 2 Department of Cardiac Anesthesiology, Sri Jayadeva Institute of Cardiovascular Sciences and Research, Bengaluru, Karnataka, India.

Dear Editor,

More than two decades ago, a fast-track protocol was contemplated by Engelman et al.^[^1^]^ for coronary artery bypass grafting (CABG) patients, at the Baystate Medical Center and Hartford Hospital. The protocol notably resulted in reduced time-to-extubation and length of stay in intensive care unit and hospital (LOS-ICU and LOS-H, respectively)^[^1^]^. Over the subsequent years, the former has increasingly been adopted in perioperative practice only to translate as an even more holistic concept of enhanced recovery after surgery (ERAS). ERAS embodies an evidence-based, multimodal, transdisciplinary care improvement initiative aimed at promoting the postoperative recovery, favorable outcome modulation, and a judicious healthcare resource utilization^[^2^]^. While the unique patient-and-procedure profle provides colossal opportunities for ERAS in cardiac surgery, accumulating parallel evidence for the effectiveness of the individual components in a bundled regimen can be challenging. With that said, multimodal analgesia is an integral consideration in conceptualizing an ERAS protocol^[^3^]^. The acute post-cardiac surgical pain of moderate-to-severe intensity often necessitates higher postoperative opioid requirements, not without the cost of potential opioid-related adverse drug events^[^4^]^. The 2019 Lancet paper by Colvin et al.^[^5^]^ delves into the debate on the opioid tolerance and hyperalgesia, suggesting accentuated opioid requirements following an escalated reliance in the early postoperative phase. On the other hand, suboptimal pain management in cardiac surgery additionally precludes an early recovery by entailing the risk of persistent postoperative pain. Therefore, there emerges a strong enough case for multimodal opioid-sparing analgesia in cardiac surgery. Indeed, the momentum surrounding non-opioid analgesia is heralded by the ERAS cardiac society class I recommendation on the formulation of a *“perioperative multimodal opioid-sparing pain management plan”*^[^2^]^.

A practical multimodal approach essentially pivots around the incorporation of effective and equally safe non-opioid pharmacological agents and regional analgesic techniques. There exists a wide range of viable non-opioid analgesics to choose from: non-steroidal anti-infammatory drugs (NSAIDs), acetaminophens, N-methyl-D-aspartate antagonists, local anesthetics, alpha-2 agonists, gabapentinoids, etc^[^4^]^. However, the extrapolation of their efficacy-safety in cardiac surgery from the array of non-cardiac perioperative research is not without its’ own faws. This can be better understood in the light of the fact that a cardiac surgical subset can be peculiarly predisposed to organ morbidity and hemodynamic instability when compared to the generalized surgical population. Thus, the distinct adverse effects (for instance, hypotension and bradycardia with alpha-2 agonists) and the potential implications of polypharmacy need to be concurrently addressed^[^4^]^. For that matter, NSAIDs administration in cardiac surgery is usually up for a debate given an intriguing literature on their accentuation of the risk of bleeding and gastrointestinal and acute kidney injury^[^4^]^. The United States Food and Drug Administration (or FDA) has issued a black-box warning for this class of drug in a setting of CABG^[^6^]^. Nonetheless, the randomized controlled trial (RCT) by Rafq et al.^[^7^]^ outlines a superior analgesia and no major organ morbidity with a multimodal dexamethasone-gabapentin-ibuprofen-paracetamol regimen in contrast to a conventional morphine-paracetamol regimen after cardiac surgery. An optimized analgesic management with avoidance of long-acting opioids and the simultaneous use of non-opioid analgesics has also been employed as an ERAS component in cardiac surgery, with encouraging results by Fleming et al.^[^8^]^.

At the same time, novel and safer regional analgesic modalities like the fascial plane blocks have motivated cardiac anesthesiologists to develop more regional-centric multimodal analgesic schemes. This paradigm shift from neuraxial-to-paraxial-to-fascial techniques in particular has settled some of the major safety concerns such as the risk of epidural hematomas amidst systemic heparinization and the possibility of hemodynamic instability with the conventional neuraxial analgesic techniques^[^4^]^. An assorted range of parasternal and chest-wall regional modalities (pectointercostal fascial block, transversus thoracis muscle plane block [TTPB], erector spinae plane block [ESPB], serratus anterior plane block [SAPB], pectoral nerves block, etc.) has received much research attention^[^9^, ^10^, ^11^, ^12^]^. While the literature is replete with the perioperative opioidsparing potential of fascial plane blocks in cardiac surgery, it only becomes imperative to reflect upon the ability to fast-track cardiac surgical cohort in background of an incorporation of the former to general anesthesia management^[^9^, ^10^, ^11^, ^12^]^. This assumes an enhanced importance given fast-tracking and ERAS are collaborative approaches in the purview of perioperative care^[^2^,^3^]^. Appropriate to the context, a reduced time-to-extubation remarkably emanated from the adult and pediatric cardiac surgery RCTs comparing the patients receiving analgesic blocks (such as ESPB and TTPB) with controls^[^9^,^10^]^. Cardinale et al.^[^11^]^ demonstrated a 50.6% operating room (OR) extubation rate owing to the inclusion of TTPB to a multimodal analgesia scheme *vs.* 8.6% OR extubation rate in the controls, in a retrospective analysis of 75 cardiac surgical patients in each group. Additionally, a reduction in LOS-ICU and LOS-H was noticed in the multimodal analgesia group in the Cardinale et al.^[^11^]^ study. Markham et al.^[^12^]^ also utilized TTPB, SAPB, and adductor canal block as key components of their ERAS protocol in CABG patients, with encouraging results.

The opioid-sparing links of multimodal analgesia ought to be nurtured in the present era of ERAS with a primary patientcentric approach of an enhanced recovery in cardiac surgery While a traditionalist might argue fddling with the position that opioids have enjoyed in the cardiac surgery, any degree of opioid stewardship materializes from evidence in ERAS (an evidence-based augmented patient recovery)^[^2^, ^3^, ^4^]^. Moreover, the cardiac surgical discipline, while awaiting more focused literature in the specialty, needs to acknowledge the vital adaptive components of ERAS, which classifes as a process rather than an outcome^[^3^]^, and the way to which can only be paved by sustainable concepts like multimodal analgesia.
